# Artificial intelligence matches subjective severity assessment of pneumonia for prediction of patient outcome and need for mechanical ventilation: a cohort study

**DOI:** 10.1038/s41598-020-79470-0

**Published:** 2021-01-13

**Authors:** Shadi Ebrahimian, Fatemeh Homayounieh, Marcio A. B. C. Rockenbach, Preetham Putha, Tarun Raj, Ittai Dayan, Bernardo C. Bizzo, Varun Buch, Dufan Wu, Kyungsang Kim, Quanzheng Li, Subba R. Digumarthy, Mannudeep K. Kalra

**Affiliations:** 1 Department of Radiology, Massachusetts General Hospital and the Harvard Medical School, 75 Blossom Court, Suite 248, Boston, MA 02114 USA; 2 MGH & BWH Center for Clinical Data Science, Boston, MA USA; 3 Employee of qure.ai, Level 6, Oberoi Commerz II, Goregaon East, Mumbai, 400063 India; 4 Gordon Center for Medical Imaging, Bartlett 501, 55 Fruit Street, Boston, MA 02114 USA

**Keywords:** Medical research, Infectious diseases

## Abstract

To compare the performance of artificial intelligence (AI) and Radiographic Assessment of Lung Edema (RALE) scores from frontal chest radiographs (CXRs) for predicting patient outcomes and the need for mechanical ventilation in COVID-19 pneumonia. Our IRB-approved study included 1367 serial CXRs from 405 adult patients (mean age 65 ± 16 years) from two sites in the US (Site A) and South Korea (Site B). We recorded information pertaining to patient demographics (age, gender), smoking history, comorbid conditions (such as cancer, cardiovascular and other diseases), vital signs (temperature, oxygen saturation), and available laboratory data (such as WBC count and CRP). Two thoracic radiologists performed the qualitative assessment of all CXRs based on the RALE score for assessing the severity of lung involvement. All CXRs were processed with a commercial AI algorithm to obtain the percentage of the lung affected with findings related to COVID-19 (AI score). Independent t- and chi-square tests were used in addition to multiple logistic regression with Area Under the Curve (AUC) as output for predicting disease outcome and the need for mechanical ventilation. The RALE and AI scores had a strong positive correlation in CXRs from each site (r^2^ = 0.79–0.86; *p* < 0.0001). Patients who died or received mechanical ventilation had significantly higher RALE and AI scores than those with recovery or without the need for mechanical ventilation (*p* < 0.001). Patients with a more substantial difference in baseline and maximum RALE scores and AI scores had a higher prevalence of death and mechanical ventilation (*p* < 0.001). The addition of patients’ age, gender, WBC count, and peripheral oxygen saturation increased the outcome prediction from 0.87 to 0.94 (95% CI 0.90–0.97) for RALE scores and from 0.82 to 0.91 (95% CI 0.87–0.95) for the AI scores. AI algorithm is as robust a predictor of adverse patient outcome (death or need for mechanical ventilation) as subjective RALE scores in patients with COVID-19 pneumonia.

## Introduction

Up to 80% of patients with single-strand ribonucleic acid (RNA), beta-coronavirus infection, also known as Coronavirus Disease of 2019 (COVID-19) are asymptomatic. In others, the disease can present with mild symptoms requiring no specific or supportive treatment to severe, life-threatening symptoms leading to acute respiratory distress syndrome, multiorgan failure, and/or thromboembolic complications^1^. Patients with severe disease often require hospitalization, mechanical ventilation, intensive care unit admission, and despite the best supportive care, some succumb to the disease. The reference diagnostic standard for COVID-19 pneumonia is the real-time reverse transcription-PCR (RT-PCR) assay using nasopharyngeal or oropharyngeal swab^2^. Imaging tests, including chest radiographs (CXRs), are not considered as diagnostic or screening tools because of low sensitivity (69% for CXR) in detecting pulmonary involvement, especially in the early stages of the disease when CXRs are often normal in patients with COVID-19 pneumonia^3,4^. CXRs can help assess severity, outcome, progression, and complications of the disease^5^.

Prior studies suggest that the Radiographic Assessment of Lung Edema (RALE) score enable assessment of the extent of pulmonary involvement in COVID-19 pneumonia and prediction of patient outcome such as hospitalization and intensive care unit (ICU) admission^6^. Artificial intelligence (AI)-based algorithms have also been reported as an accurate method for detecting the severity of lung involvement and distinguishing between moderate and severe pneumonia on CXRs^7^. AI algorithms were also sensitive for differentiating bacterial and other viral pneumonias from COVID-19 pneumonia^8^. Most AI studies focus on the diagnosis of COVID-19 pneumonia on baseline CXRs, with only a few studies on the role of AI for predicting disease progression and patient outcomes on the baseline and/or serial CXRs. We processed multicenter CXRs with a commercially available AI algorithm (qXR v2.1 c2, Qure.ai Technologies, Mumbai, India), which was specifically trained with data from patients with RT-PCR assay positive COVID-19. The algorithm provides a pixel level border and percentage of projected lung area affected with COVID-19 related findings. The purpose of our study was to compare the performance of AI and RALE scores from CXRs for predicting patient outcomes and the need for mechanical ventilation in COVID-19 pneumonia.

## Methods

We performed an Institutional Review Board (IRB) approved (Partners Human Research Committee, Protocol #: 2016P000767/PHS), HIPAA compliant study with a waiver of informed consent. All methods were carried out in accordance with relevant guidelines and regulations.

### Patients

Our study included 405 adult patients from the United States (Site A: n = 226 patients at Massachusetts General Hospital, Boston MA) and South Korea (Site B: n = 179 patients from affiliated hospitals in Daegu, South Korea including Kyungpook National University, Yeungnam University College of Medicine, Keimyung University School of Medicine and Catholic University of Daegu School of Medicine). All participating hospitals were tertiary care hospitals.

The inclusion criteria for the study were RT-PCR positive COVID-19 pneumonia, availability of CXRs, and patient outcome data such as death or recovery from COVID-19 infection and mechanical ventilation. All CXRs from the onset of symptoms or RT-PCR testing were included in the study. Patients with artifacts and low quality CXRs (incompletely imaged lungs) were excluded. A total of 1367 CXRs were included in the study with 644 CXRs from Site A (1–11 CXRs/patient) and 723 CXRs from Site B (1–20 CXRs/patient).

We reviewed patients’ electronic medical records to obtain information pertaining to their demographics (age, gender, body mass index -BMI), smoking history, comorbid conditions (such as cardiovascular diseases, blood disorders, kidney diseases, liver diseases, respiratory diseases, metabolic syndromes, and neurodegenerative disorders from Site A only), vital signs (body temperature and peripheral oxygen saturation at the time of admission) and laboratory data (including white blood cell count, platelet count, and C-reactive protein- CRP from Site B only).

### Qualitative assessment

**Figure 1:**
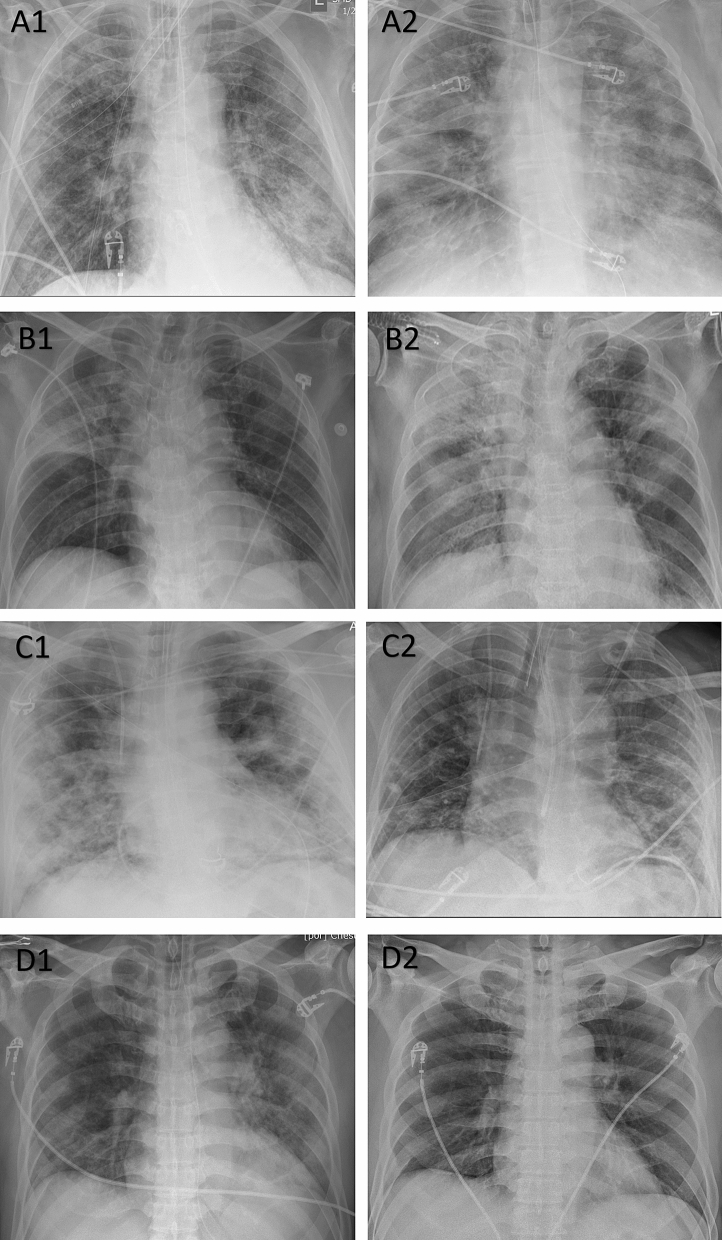
Frontal CXRs of patients with RT-PCR positive COVID-19 pneumonia. **A** A 65-year-old male from Site A with baseline (A1: RALE score 12, AI score 101) and follow-up CXRs (A2: RALE score 32, AI score 175) received mechanical ventilation and died after 30 days of hospitalization. **B** An 84-year-old male from Site B with baseline (B1: RALE score 10, AI score 59) and follow-up CXRs (B2: RALE score of 29, AI score 127) received mechanical ventilation and expired after 17 days of hospitalization. **C** A 58-year-old male from Site A had full recovery following hospitalization (baseline CXR, C1: RALE score 24, AI score 156; follow-up CXR, C2: RALE score 9, AI score 133). The patient required mechanical ventilation. **D** A 57-year-old male from Site B had full recovery following hospitalization. The radiographic opacities on baseline CXR (D1: RALE score 11, AI score 98) resolved on follow-up CXR (D2: RALE score 0, AI score 0). The patient did not require mechanical ventilation.

Two thoracic radiologists (SRD—16 years of experience; MKK—13 years of experience) performed the qualitative assessment of all de-identified frontal CXRs included in the study. They used the previously described RALE score for assessing the radiographic extent and the severity of lung involvement from COVID-19 pneumonia^9^. For the RALE score, each lung was divided into two quadrants (upper and lower quadrants) by a vertical line through the vertebral column and a horizontal line at the level of the origin of the upper lobe bronchus from the left main bronchus. Within each quadrant, opacities were separately scored for density (scores 1, 2 and 3 for hazy, intermediate and dense consolidation, respectively) and extent (scores 0, 1, 2, 3, and 4 for none, < 25%, 25–50%, 50–75%, and > 75% of quadrant involved, respectively) (Fig. 1). RALE score represented the sum of the products of density and extent scores of each lobe (minimum score 0; maximum score 48). As a surrogate of approximate lung volume, we recorded the number of right anterior rib, which crossed the anterior aspect of the right hemidiaphragm.

### AI algorithm

DICOM images of all frontal CXRs were imported into a commercial AI algorithm (qure.ai, Mumbai, India) and processed by two study coauthors (SE and FH with 1-year post-doctoral research experience in thoracic imaging). The algorithm provides the percentage of projected area with COVID-19 related findings which we deemed as the AI score. The processing time per CXR was < 5 s.

The AI algorithm is a deep learning-based model trained with two sets of data. With the first set of 2.5 million CXRs, the algorithm was trained and validated for detection and distribution of pulmonary opacities along with presence of other radiographic findings such as hilar enlargement, pleural effusions, cavities, nodules, and calcifications. The second set of 600 CXRs (300 CXRs from RT-PCR assay positive COVID-19 positive patients and 300 CXRs without COVID-19 pneumonia) were used to train the algorithm to output COVID-19 prediction scores. None of the two datasets belonged to any of the participating institutions or countries included in our study.

The abnormality detection AI algorithm in our study is composed of two parts. First, the abnormality-specific region of interest (ROI) generator comprises multiple segmentation networks using U-Net architecture^10^. It creates a mask for different anatomies such as lungs, diaphragm, and mediastinum and then generates a set of ROIs with a specific abnormality. Second, a hybrid convolutional neural network generates outputs of a low-resolution probability map and a prediction score of findings. The predictions from each of the multiple ROIs are pooled with the Log-Sum-Exp function (a convex approximation of the maximum function) to obtain the overall prediction score and pixel map^11–13^. The hybrid network was trained end-to-end using both Natural Language Processing-inferred labels from radiology reports and pixel-level annotations from radiologists where available.

Upon completion of processing, the AI algorithm outputs a secondary capture DICOM with the following components: pixel-level border (the affected lung regions with COVID-19 related findings), percentage of projected area with COVID-19 related findings, the risk of the CXR being from COVID-19 positive patient (COVID-19 risk as high, medium, low and none) and a COVID-19 score for each lung, separately. The total AI score was estimated by adding scores for each lung.

### Code availability

The Qure.ai algorithm used in our study is commercially available for clinical use in Europe (CE approved). At the time of writing of this manuscript the AI algorithm was not approved for clinical use in the US. Users can try the algorithm on the vendor website (https://scan.qure.ai/ accessed on 11.18.2020).

### Statistical analyses

The RALE and AI scores from the baseline and serial CXRs were recorded in Microsoft EXCEL (Microsoft Inc., Redmond, Washington, USA). For patients with multiple CXRs, we estimated the maximum RALE and AI scores. Independent t- and chi-square tests were used to analyze the quantitative and qualitative variables, respectively. We obtained odd’s ratio (OR) to predict patient outcomes from different clinical and laboratory variables. We used a linear correlation test to estimate the direction and magnitude of the association between the RALE and AI scores. We estimated the percentage agreement between the trends of AI and RALE scores over serial CXRs. Multiple logistic regression was performed with Area Under the Curve (AUC) as output for predicting disease outcome and the need for mechanical ventilation (SPSS version 24, IBM, Chicago, IL). A p-value of less than 0.05 was deemed to suggest a significant statistical difference.

## Results

### Clinical and laboratory information

The mean age (and standard deviations) of patients from sites A and B were 65 ± 16 years (age range 23–96 years) and 63 ± 17 years (age range 20–97 years), respectively.

Among the 405 patients from both sites, 147 patients died (Site A: 98/226, Site B: 49/179) and 258 patients recovered (Site A: 128/226, Site B: 130/179) from COVID-19 pneumonia. Of these, 124 patients (Site A: 92/226, Site B: 32/179) required mechanical ventilation during hospitalization. At both sites, patients who died were significantly older than those who recovered from their infection (*p* < 0.001). Age was associated with a higher risk of mortality (AUC up to 0.78), but not the need for mechanical ventilation (AUC up to 0.56). The demographic data for the patients from both sites are summarized in Tables 1, 2, 3 and 4.

**Table 1:** Summary of assessed variables for prediction of death versus recovery from COVID-19 pneumonia in patients from Site A.

	Outcome		*p* value	AUC (95% CI) [OR]
	Death	Recovery		
	(n = 98)	(n = 128)		
Age (mean ± SD, years)	73 ± 12	60 ± 17	< 0.001	0.70 (0.61–0.78)
Male/Female	56/42 [57%/43%]	79/49 [62%/38%]	0.549	[0.84]
Smoking (Y/N)	15/83 [15%/85%]	9/119 [7%/93%]	0.045	[2.4]
Cancer (Y/N)	30/68 [31%/69%]	17/111 [13%/87%]	0.001	[2.9]
Cardiovascular ds (Y/N)	59/39 [60%/40%]	61/67 [48%/52%]	0.061	[1.7]
Blood ds (Y/N)	11/87 [11%/89%]	9/119 [7%/93%]	0.271	[1.7]
Kidney ds (Y/N)	15/83 [15%/85%]	10/118 [8%/92%]	0.075	[2.1]
Liver ds (Y/N)	4/94 [4%/96%]	8/120 [6%/94%]	0.471	[0.6]
Metabolic ds (Y/N)	56/42 [57%/43%]	67/61 [52%/48%]	0.473	[1.2]
Neurodegenerative ds (Y/N)	14/84 [14%/86%]	5/123 [4%/96%]	0.005	[4.1]
Lung ds (Y/N)	28/70 [29%/71%]	24/104 [19%/81%]	0.082	[1.7]
Baseline RALE	8.9 ± 8.9	3.7 ± 5.2	< 0.001	0.71 (0.64–0.78)
Max RALE	18.9 ± 8.6	7.9 ± 7.7	< 0.001	0.83 (0.77–0.90)
Baseline AI score	68.2 ± 48.4	43.2 ± 45.9	< 0.001	0.66 (0.58–0.73)
Max AI score	121.0 ± 34.6	83.8 ± 53.9	< 0.001	0.71 (0.62–0.80)
RALE score change	10.6 ± 8.7	3.5 ± 5.1	< 0.001	0.75 (0.68–0.83)
AI score change	54.9 ± 47.1	31.8 ± 38.0	0.001	0.65 (0.56–0.74)

Maximum RALE scores were stronger predictors than AI scores for final patient outcome.

*SD* standard deviation, *Y/N* present or absent, *ds* disease, *max* maximum, *AUC* area under the curve, *CI* confidence interval, *OR* odd’s ratio, *%* percentage.

Numbers in square parenthesis represent odd’s ratios (R2-14).

**Table 2:** Summary of assessed variables for predicting need for mechanical ventilation in COVID-19 patients from Site A.

	Mechanical ventilation		*p* value	AUC (95% CI) [OR]
	Yes	No		
	(n = 92)	(n = 134)		
Age (mean ± SD, years)	65 ± 15	66 ± 18	0.955	0.52 (0.45–0.60)
Male/Female	60/32 [65%/35%]	75/59 [56%/44%]	0.155	[1.5]
Smoking (Y/N)	12/80 [13%/87%]	12/122 [9%/91%]	0.332	[1.5]
Cancer (Y/N)	21/71 [23%/77%]	26/108 [19%/81%]	0.543	[1.2]
Cardiovascular ds (Y/N)	45/47 [49%/51%]	75/59 [56%/44%]	0.278	[0.7]
Blood ds (Y/N)	8/84 [9%/91%]	12/122 [9%/91%]	0.904	[1.1]
Kidney ds (Y/N)	10/82 [11%/89%]	15/119 [11%/89%]	0.931	[0.9]
Liver ds (Y/N)	1/91 [1%/99%]	11/123 [8%/92%]	0.019	[1.5]
Metabolic ds (Y/N)	45/47 [49%/51%]	78/56 [58%/42%]	0.190	[0.7]
Neurodegenerative ds (Y/N)	4/88 [4%/96%]	15/119 [11%/89%]	0.067	[0.3]
Lung ds (Y/N)	21/71 [23%/77%]	31/103 [23%/77%]	0.944	[0.9]
Baseline RALE	8.6 ± 9.1	4.1 ± 5.5	< 0.001	0.66 (0.59–0.74)
Max RALE	17.2 ± 9.1	10.9 ± 9.6	< 0.001	0.70 (0.62–0.78)
Baseline AI score	69.6 ± 48.9	43.4 ± 45.7	< 0.001	0.66 (0.58–0.73)
Max AI score	122.9 ± 38.0	86.9 ± 49.6	< 0.001	0.70 (0.62–0.78)
RALE score change	9.1 ± 8.2	5.8 ± 7.7	0.013	0.63 (0.54–0.72)
AI score change	57.3 ± 45.4	32.4 ± 40.7	0.001	0.67 (0.58–0.76)

Maximum RALE and AI scores were the strongest predictors.

*SD* standard deviation, *Y/N* present or absent, *ds* disease, *max* maximum, *AUC* area under the curve, *CI* confidence interval, *OR* odd’s ratio, *%* percentage.

Numbers in square parenthesis represent odd’s ratios (R2-14).

**Table 3:** Summary of assessed variables for prediction of death versus recovery from COVID-19 pneumonia in patients from Site B.

	Outcomes		*p* value	AUC (95% CI) [OR]
	Death	Recovery		
	(n = 49)	(n = 130)		
Age (mean ± SD, years)	75 ± 9	59 ± 17	< 0.001	0.78 (0.69–0.86)
Male/Female	31/18 [63%/37%]	57/73 [44%/56%]	0.020	[2.2]
BMI	24.4 ± 4.1	23.8 ± 2.7	0.423	0.55 (0.41–0.70)
SpO2	94.1 ± 8.2	97.0 ± 3.1	0.018	0.48 (0.32–0.64)
Temperature (Celsius)	36.9 ± 0.7	36.9 ± 0.7	0.788	0.49 (0.35–0.62)
Total WBC count	8911.1 ± 4349.1	5853.7 ± 2094.1	< 0.001	0.76 (0.64–0.89)
Platelet count	205.1 ± 97.8	229.2 ± 87.7	0.120	0.37 (0.24–0.50)
CRP	25.2 ± 31.8	16.9 ± 28.2	0.103	0.68 (0.59–0.77)
Baseline RALE	15.6 ± 8.4	4.1 ± 6.2	< 0.001	0.87 (0.81–0.93)
Max RALE	29.8 ± 10.1	6.3 ± 7.9	< 0.001	0.95 (0.91–0.99)
Baseline AI score	100.6 ± 39.8	40.7 ± 47.8	< 0.001	0.82 (0.76–0.89)
Max AI score	149.2 ± 25.3	61.4 ± 55.6	< 0.001	0.91 (0.86–0.96)
RALE score change	13.9 ± 10.2	2.3 ± 4.2	< 0.001	0.86 (0.79–0.93)
AI score change	45.6 ± 37.1	20.6 ± 26.8	< 0.001	0.72 (0.63–0.81)

Maximum RALE and AI scores were the strongest predictors of final patient outcome.

*SD* standard deviation, *BMI* body mass index, *SpO*_*2*_ peripheral oxygen saturation, *CRP* c-reactive protein, *max* maximum, *AUC* area under the curve, *CI* confidence interval, *OR* odd’s ratio, *%* percentage.

Numbers in square parenthesis represent odd’s ratios (R2-14).

**Table 4:** Summary of assessed variables for predicting need for mechanical ventilation in patients from Site B.

	Mechanical ventilation		*p* value	AUC (95% CI) [OR]
	Yes	No		
	(n = 32)	(n = 147)		
Age (mean ± SD, years)	66.7 ± 10.3	62.1 ± 17.9	0.056	0.56 (0.48–0.65)
Male/Female	20/12 [62%/38%]	68/79 [46%/54%]	0.096	[1.9]
BMI	25.7 ± 3.5	23.5 ± 2.7	< 0.001	0.64 (0.51–0.77)
SpO2	93.0 ± 9.5	96.9 ± 3.3	0.031	0.41 (0.25–0.56)
Temperature (Celsius)	37.1 ± 0.7	36.9 ± 0.7	0.195	0.55 (0.42–0.68)
Total WBC count	9135.6 ± 4266.1	6160.5 ± 2632.2	0.001	0.75 (0.62–0.87)
Platelet count	217.7 ± 83.6	223.9 ± 92.7	0.729	0.53 (0.39–0.67)
CRP	23.0 ± 25.8	18.3 ± 30.1	0.429	0.71 (0.61–0.80)
Baseline RALE	15.2 ± 9.1	5.5 ± 7.4	< 0.001	0.81 (0.73–0.88)
Max RALE	27.7 ± 9.9	9.0 ± 11.5	< 0.001	0.88 (0.83–0.93)
Baseline AI score	100.9 ± 38.1	47.6 ± 50.9	< 0.001	0.79 (0.71–0.86)
Max AI score	145.7 ± 24.1	70.5 ± 60.6	< 0.001	0.84 (0.79–0.90)
RALE score change	12.5 ± 10.1	3.7 ± 6.6	< 0.001	0.79 (0.70–0.88)
AI score change	44.7 ± 38.7	23.1 ± 28.6	< 0.001	0.68 (0.57–0.78)

Maximum RALE and AI scores were the strongest predictors of need for mechanical ventilation.

*SD* standard deviation, *BMI* body mass index, *SpO*_*2*_ peripheral oxygen saturation, *CRP* c-reactive protein, *max* maximum, *AUC* area under the curve, *CI* confidence interval, *OR* odd’s ratio, *%* percentage.

Numbers in square parenthesis represent odd’s ratios (R2-14).

Patients with smoking history, as well as the presence of neurodegenerative disorders and cancer, were more common in patients who died from COVID-19 pneumonia as compared to patients who survived (*p* = 0.001–0.045). Patients who needed mechanical ventilation had higher rates of liver disorders than those who did not require mechanical ventilation (*p* = 0.019).

History of cancer (OR 2.9, 95% confidence interval (CI) 1.5–5.6) and neurodegenerative diseases (OR 4.1, 95% CI 1.4–11.8) were independent predictors of mortality from COVID-19 pneumonia.

Total WBC count (AUC 0.76, 95% CI 0.64–0.89) and peripheral oxygen saturation < 93% (OR 5.7, 95% CI 2.4–13.8) were strong predictors of death-related to COVID-19 pneumonia. Both the total WBC counts (AUC 0.75, 95% CI 0.62–0.87) and oxygen saturation < 93% (OR 4.5, 95% CI 1.8–11.2) were independent predictors of mechanical ventilation. Other clinical and laboratory data, including the CRP or platelet counts, were not associated with a higher rate of mortality or mechanical ventilation (*p* = 0.103–0.729) (Tables 1, 2, 3 and 4).

### RALE and AI scores

The RALE and AI scores had a strong positive correlation in the entire datasets (r^2^ = 0.83, *p* < 0.0001) as well as at the level of each participating site (Site A: r^2^ = 0.79, *p* < 0.0001; Site B: r^2^ = 0.86, *p* < 0.0001). There was a strong percentage agreement between the changes over serial CXRs for RALE and AI scores from both sites (Site A: 75.3%; Site B: 77.1%).

Both the baseline and maximum RALE and AI scores in patients who died or received mechanical ventilation were significantly higher than the corresponding scores than those with recovery or without need for mechanical ventilation (p < 0.001–0.013) (Tables 1, 2, 3 and 4). Among patients with serial CXRs (n = 323/405), those who died and received mechanical ventilation had significantly greater RALE and AI score changes (*p* < 0.001–0.013) (Tables 1, 2, 3 and 4).

**Figure 2:**
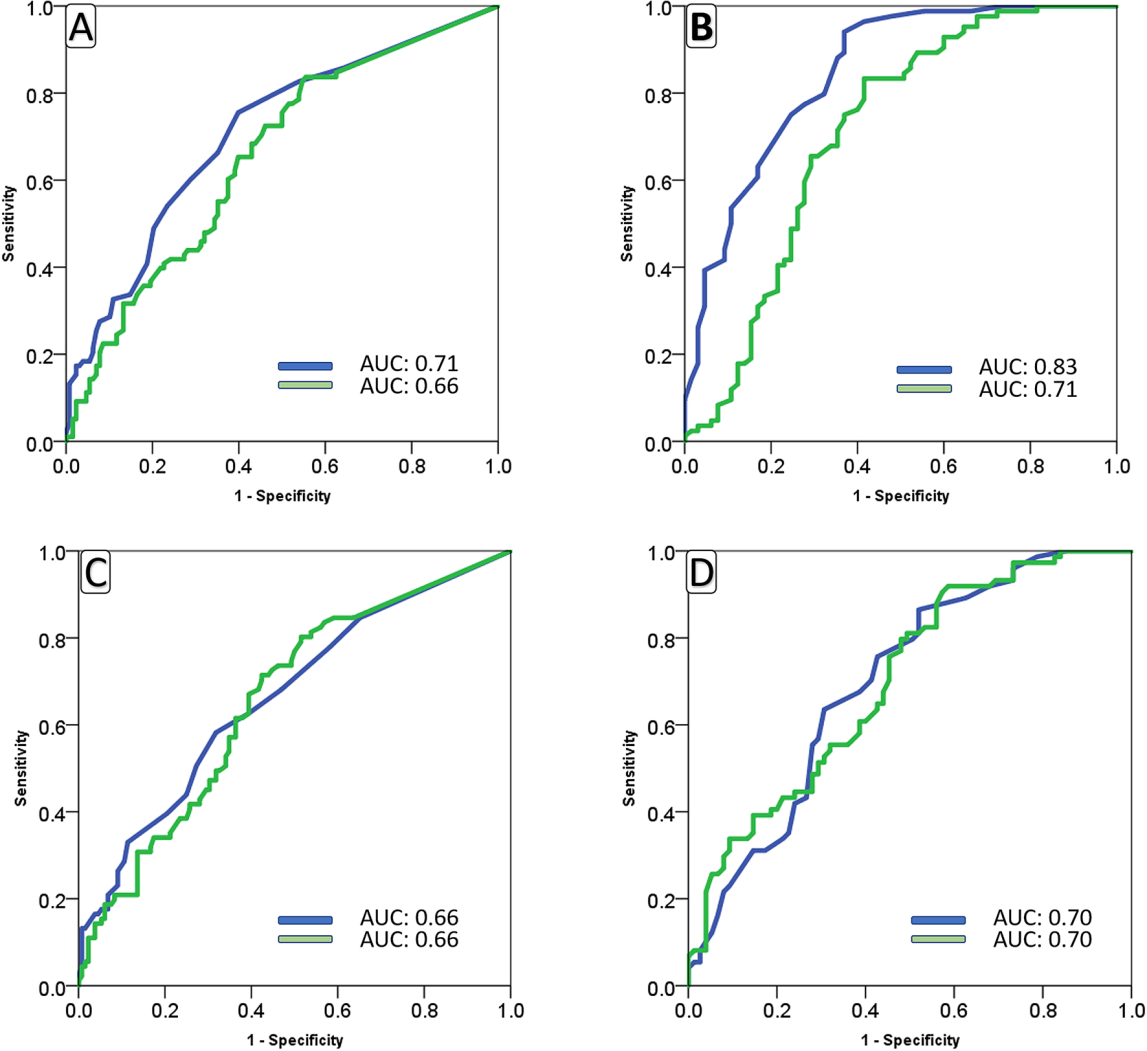
Area under the curve for baseline (**A**,**C**) and maximum (**B**,**D**) RALE (blue) and AI (green) scores in Site A patients with different outcomes (**A**,**B**) of COVID-19 infection and need for mechanical ventilation (**C**,**D**).

Figures 2 (Site A) and 3 (Site B) summarize the site-specific performance of RALE and AI scores. Table 5 summarizes the best sensitivity and specificities for RALE and AI scores for prediction of death and mechanical ventilation.

**Figure 3:**
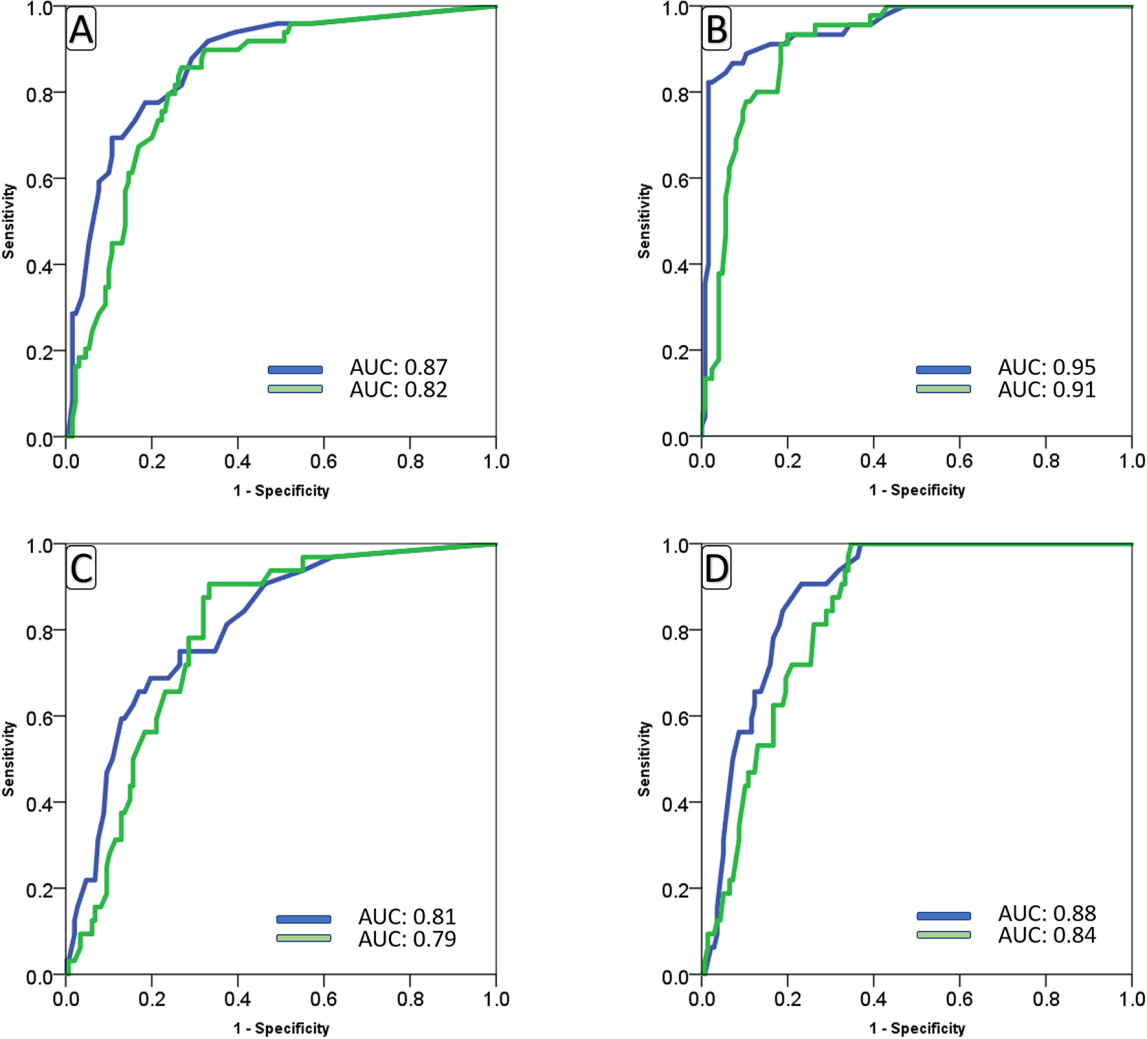
Area under the curve for baseline (**A**,**C**) and maximum (**B**,**D**) RALE (blue) and AI (green) scores in Site B patients with different outcomes (**A**,**B**) of COVID-19 infection and need for mechanical ventilation (**C**,**D**).

**Table 5:** Site-specific thresholds of RALE and AI scores with the best sensitivities and specificities for different outcomes.

	Site A		Site B	
	Mechanical ventilation	Outcome	Mechanical ventilation	Outcome
Best sensitivities (RALE)				
Threshold RALE score	21	7	29	18
Sensitivity	90%	95%	90%	89%
Specificity	30%	63%	55%	81%
Best sensitivities (AI)				
Threshold AI score	135	53	98	143
Sensitivity	90%	96%	97%	90%
Specificity	40%	52%	66%	67%
Best specificities (RALE)				
Threshold RALE score	6	23	11	22
Sensitivity	55%	33%	68%	77%
Specificity	89%	91%	91%	98%
Best specificities (AI)				
Threshold AT score	54	60	106	106
Sensitivity	52%	55%	67%	73%
Specificity	89%	90%	91%	90%

There was no difference in prediction of COVID-19 mortality from baseline CXR’s RALE score (AUC 0.87) and the maximum difference between RALE scores across serial CXRs (AUC up to 0.86) (*p* > 0.05). However, AI scores from the baseline CXR (AUC 0.82) were better predictors of patient outcome than the changes in AI scores over serial CXRs (AUC up to 0.72) (*p* < 0.05).

In site A, a combination of baseline RALE and AI score with patients’ age and smoking history increased the outcome prediction from 0.71 to 0.80 (95% CI 0.75–0.86) for RALE score and from 0.66 to 0.78 (95% CI 0.73–0.84) for AI score. In site B, a combination of either baseline RALE or AI scores with patients’ age, gender, WBC count, and peripheral oxygen saturation increased the outcome prediction from 0.87 to 0.94 (95% CI 0.90–0.97) for RALE scores and from 0.82 to 0.91 (95% CI 0.87–0.95) for the AI scores. The addition of age, BMI, WBC count, and peripheral oxygen saturation to the RALE score and AI score increased the accuracy for predicting the need for mechanical ventilation to 0.89 (95% CI 0.82–0.96) for RALE score and 0.90 (0.85–0.95) for AI score.

### Lung volume measurements

The lung volumes across the two sites were not significantly different (*p* = 0.162). In CXRs from site A, the right hemidiaphragm position was similar in patients who died or received mechanical ventilation (anterior right rib level: 5.1 ± 0.9) compared to those with favorable outcomes (5.0 ± 0.6) (*p* = 0.601).

At site B, the level of right hemidiaphragm was slightly but significantly higher in patients who died (anterior right rib level of 5.0 ± 0.7) than in those with recovery (5.5 ± 0.7) (*p* < 0.001). There was no significant difference in the level of right hemidiaphragm in patients who needed mechanical ventilation (5.2 ± 0.7) versus those who did not (5.5 ± 0.7) (*p* = 0.053).

## Discussion

We found that both RALE and AI scores derived from CXRs can predict the need for mechanical ventilation and death in patients with COVID-19 pneumonia. Strong correlation between RALE and AI scores in our study (r^2^ = 0.79–0.86) is similar to a recent study from Cohen et al. (r^2^ = 0.81–0.83)^14^. In a recent study on 697 patients with COVID-19 pneumonia with the same Qure.ai algorithm, the AI score was reported as an independent predictor of patients’ outcome^15^. Although our results are consistent with recent CXRs studies with both RALE and AI algorithm-generated severity assessment^14,16^, there are some notable differences. As opposed to prior studies on baseline CXRs at hospital admission^6^, we assessed the performance of severity assessment on serial CXRs. Maximum RALE or AI scores in follow-up CXRs rather than previously reported scores on baseline CXRs^6,16^ were stronger predictors of assessed outcome variables in our study. Changes in RALE and AI scores over serial CXRs, not assessed in prior publications, were predictive of patient outcomes. As opposed to standalone interpretation and reporting of CXRs findings, we also found that the addition of clinical and laboratory information into regression models significantly improves their predictive value. Although lung volumes in patients with adverse outcomes tend to be lower than in those with favorable outcome, the difference were not statistically significant. Reduced ventilatory capacity in adverse outcome patients with advanced or more severe disease likely explains the differences in lung volumes noted in our study.

Another new information pertains to the differences in the absolute severity of radiographic findings in patients with favorable and unfavorable outcomes at the two participating sites included in our study. The maximum and baseline RALE and AI scores at Site A were lower than those on CXRs from Site B in patients who died or needed mechanical ventilation. However, both scores from either site in our study were higher than those reported from deceased patients in a prior study (mean RALE of 14) from Italy^17^. Although our study did not assess the cause of variations in severity scores, there could be several reasons for this finding. Technical differences in CXRs (such as the distribution of digital versus conventional CXRs and portable anteroposterior versus upright posteroanterior projection CXRs) can influence the attenuation of radiographic opacities and lead to variations in perceived and quantitative severity of pulmonary involvement. Although technical and patient factors can lead to differences in lung volumes, this was unlikely a substantial contributor since lung volumes estimated from the level of the right hemidiaphragm were similar across the two sites in our study. Differences in patient size across different sites can also lead to differences in CXR image quality and affect severity assessment. Besides these factors, the differences in severity scores across various sites could also be related to patient death or the need for mechanical ventilation from non-pulmonary complications or other underlying comorbidities. Differences in supportive treatment strategies at the participating sites could also be responsible for variations in radiographic severity scores. These technical and patient-related reasons might also explain the differences in performance of both RALE and AI scores at the two participating sites (AUC for prediction of mechanical ventilation at Site B was better than at Site A).

Although most imaging and AI literature focus on the use of chest CT for assessing severity, complications^18^, and outcome in patients with COVID-19 infection, the main implication of our study lies in the use of CXRs as a powerful tool to assess disease severity and predict patient outcomes. Apart from being substantially lower in radiation dose compared to most chest CT protocols, CXRs units are more portable, easy to sterilize, rapid, accessible, and available in the emergency rooms and by the bedside. Apart from the pulmonary opacities, CXRs help assess lines and tubes which need frequent confirmation for placement in critically ill patients such as endotracheal tubes, esophageal tubes, central lines and other life support catheters.

Another implication pertains that compared to the RALE score, AI scores are quantitative, rapid, automated, and least disruptive to the workflow of CXRs’ interpretation. Prediction of information pertaining to the need for mechanical ventilation and the likelihood of adverse outcomes can help manage the patient and anticipate the resources needed for patient care in a high prevalence disease setting. Future prospective studies will be required to answer the crucial questions on the impact of such predictive information on patient care and resource planning.

There are limitations to our study. First, our study was a retrospective prediction of patients with known outcomes. We minimized bias by ensuring that the radiologists participating in RALE score assignment or investigators processing the CXRs with the AI algorithm were not aware of the patient outcomes before completing the data collection and image analyses. Second, we did not perform the statistical power of our study and instead included all subjects who met our inclusion criteria. Third, we did not have access to all clinical and laboratory data variables from both study sites. Fourth, we did not normalize the data for the effects of variable use of management strategies (such as prophylactic anticoagulation and clinical drug trials) on the prediction of outcome based on either scoring systems. We did not assess if RALE or AI scores can help predict or evaluate treatment response of definitive or supportive treatment in our patients. Since identifying data from the four participating sites in South Korea were randomized and deidentified to protect patient privacy, it was not possible to compare patient characteristics between the four sites or to find if some patients were transferred between different hospitals following their baseline CXRs.

Fifth, we did not have access to information between the onset of patients’ symptoms or RT-PCR assay and the baseline CXR. However, despite the lack of such information, both RALE and AI scores had high AUCs for predicting mortality and need for mechanical ventilation. Although the AI algorithm was generalizable at both participating institutions, we did not assess its broader generalizability in other institutions and/or regions. We did not determine the accuracy of localization or severity of pulmonary opacities by the AI algorithm. However, a strong correlation with RALE score and similar performance as RALE score provide evidence of its accuracy. Moreover, prior studies with the same algorithm have reported the accuracy of the algorithm in non-COVID patients^19^. Finally, we did not compare the performance of our AI algorithm with other algorithms.

In summary, the severity score from the AI algorithm is as robust a predictor of adverse patient outcome (death or need for mechanical ventilation) as subjective RALE scores in patients with COVID-19 pneumonia. Maximum RALE and AI scores over serial CXRs were more reliable predictors of the patient outcome than scores from baseline CXRs. The addition of clinical and laboratory information improves the performance of both the RALE and the AI scores.

## Abbreviations

AI: Artificial intelligence
CXR: Chest radiographs
IRB: Institutional Review Board
WBC: White blood cell
CRP: C-reactive protein
RALE: Radiographic Assessment of Lung Edema
AUC: Area under the curve
RT-PCR: Reverse transcription-polymerase chain reaction
ICU: Intensive care unit
HIPAA: Health Insurance Portability and Accountability Act
BMI: Body Mass Index
ROI: Region of Interest
DICOM: Digital imaging and communications in medicine
CI: Confidence interval
OR: Odds ratio
